# Metachronous Multifocal Extradigital Glomus Tumor of Lower Extremity: A Case Report and Literature Review

**DOI:** 10.1155/cro/1411715

**Published:** 2026-07-24

**Authors:** Giulia De Marco, Giuseppe Francesco Papalia, Luisana Sisca, Francesco Luigi Gambaro, Bruno Vincenzi, Nicola Papapietro

**Affiliations:** ^1^ Research Unit of Orthopaedic and Trauma Surgery, Department of Medicine and Surgery, Università Campus Bio-Medico di Roma, Rome, Italy, unicampus.it; ^2^ Operative Research Unit of Orthopaedic and Trauma Surgery, Fondazione Policlinico Universitario Campus Bio-Medico, Rome, Italy; ^3^ Oncological Orthopaedics Department, IFO-IRCCS Regina Elena National Cancer Institute, Rome, Italy; ^4^ Department of Medical Oncology, Fondazione Policlinico Universitario Campus Bio-Medico, Rome, Italy; ^5^ Anatomical Pathology Operative Research Unit, Fondazione Policlinico Universitario Campus Bio-Medico, Roma, Italy

**Keywords:** case report, extradigital, glomus tumor, late metachronous, literature review, recurrence

## Abstract

The glomus tumor (GT) is a rare benign neoplasm, generally located in the subungual region of the distal phalanx of the fingers as a single nodule. While approximately 90% of digital GTs are correctly diagnosed (pain, cold sensitivity, and point tenderness), extradigital forms often present with silent or atypical features, complicating diagnosis. The treatment of choice is surgical excision, associated with low recurrence rates. In October 2022, a 64‐year‐old man presented with a painful, palpable mass in the distal third of the right femur. Magnetic resonance and computed tomography revealed a solid lesion involving the distal third of the femur. A needle biopsy confirmed the diagnosis of GT with hemangiopericytoma‐like features. In January 2023, surgical excision was performed, and histology confirmed the diagnosis. The patient had a significant oncologic history: In 1994, he underwent wide resection and adjuvant chemotherapy for a high‐grade undifferentiated sarcoma of the right thigh. In November 2012, he reported a painful mass in the right ankle, and excisional biopsy with immunohistochemistry led to a diagnosis of multifocal GT. At his last follow‐up in November 2025, he was in good general health under oncologic surveillance. We conducted a search on PubMed and Scopus using the keywords “extra digital glomus tumor” and included articles reporting extra digital GTs of the lower limb. The literature review identified 143 patients with extradigital GT with lower limb involvement. This case represents a rare presentation because of its unusual lower limb location, with lesions in the ankle in 2012 and the femur in 2022. We report a rare case of a late metachronous extradigital GT of the lower limb in an unusual anatomical site.

## 1. Introduction

Glomus tumor (GT) is a rare benign perivascular neoplasm that originates from modified smooth muscle cells of the glomus body. According to the WHO classification of soft tissue tumors, the GT is classified as a pericytic neoplasm together with myopericytoma/myofibroma and angioleiomyoma [1]. Although the exact etiopathogenesis of the GT remains unclear, both hereditary factors and prior traumatic events have been implicated as potential contributors. Specifically, mechanical trauma may induce local proliferation of glomus bodies, whereas a potential genetic predisposition is supported by its documented association with Neurofibromatosis Type 1 [2–4]. These uncommon tumors represent a small subset of soft tissue tumors (up to 6%) and a comparable proportion of all hand neoplasms (1%–5%) [5]. Most lesions are small (< 1 cm), solitary, and located in the subungual region of the fingers, whereas extradigital presentations are uncommon and may involve atypical sites throughout the body, with the forearm being the most frequently reported extradigital site [2, 6, 7]. Recent studies also suggest that extradigital GTs are more common than is frequently assumed [8]. Clinically, digital GTs typically present with the triad of pain, cold sensitivity, and point tenderness; however, these clinical findings may be absent in extradigital lesions, making diagnosis more difficult and leading to diagnostic delay [6, 7, 9]. In atypical locations, GTs can remain silent for a long time until an incidental trauma elicits symptoms. While almost all digital GTs are correctly identified through physical examination alone, their extradigital counterparts are commonly misdiagnosed; in fact, recent data indicate that only one in five atypical presentations is correctly diagnosed [10–12]. The epidemiological distribution of extradigital GTs differs notably from classic digital lesions. Digital GTs are approximately twice as common in women; conversely, extradigital lesions show a male predominance with a male–female ratio greater than 4:1 and tend to occur in older patients. Additionally, the characteristic pain and hypersensitivity to cold are less prevalent in these atypical locations [6, 9, 10]. Although part of the same pathological entity, digital and extradigital GTs present distinct clinical and epidemiological features. The clinical suspicion of a GT is generally supported by ultrasound (US) and, if necessary, by magnetic resonance imaging (MRI) for accurate lesion localization and assessment of its margins; however, histopathological examination remains essential for definitive diagnosis [13]. Ultrasonography typically demonstrates a small (≤ 1.0 cm), solid, well‐demarcated nodule that is homogeneously hypoechoic or isoechoic, with hypervascularity on color or power Doppler imaging, and possible bony erosion of the underlying phalangeal bone [11]. On MRI, the lesion is usually well‐marginalized, oval, and hypointense on T1‐weighted sequences, whereas it appears hyperintense on T2‐weighted imaging, often surrounded by a hypointense rim [4]. Histologically, a GT is a well‐demarcated neoplasm characterized by a solid nodular proliferation of uniform, rounded to spindled cells intimately associated with dilated, branching, and thickened vascular channels. The neoplastic glomus cells show a syncytial growth pattern, pale to eosinophilic cytoplasm, and centrally located round to ovoid nuclei without significant pleomorphism or mitotic activity. The stroma may show variable hyalinization and myxoid change, often forming nests of cells in a solid or trabecular arrangement. Immunohistochemical analysis confirms the perivascular myoid differentiation, showing diffuse positivity for alpha‐smooth muscle actin (SMA) and h‐caldesmon, whereas CD34, S100, and cytokeratins are negative [14, 15]. Complete surgical excision is the treatment of choice for isolated GTs and is generally associated with excellent symptom relief and low recurrence rates [2, 11]. Recurrences occurring at the postoperative second or third year are usually attributable to multiple primary tumors [16]. We conducted a comprehensive literature review focused on extradigital GTs, with particular attention to lower limb localization, to evaluate their overall incidence, anatomical distribution, clinical manifestation, surgical management strategies, postoperative outcomes, and long‐term recurrence rate. Furthermore, we report a rare case of an atypical extradigital GT of the lower extremity in a male patient with involvement of two distinct anatomical sites over a 10‐year period.

## 2. Case Presentation

A 64‐year‐old male presented to our institution in October 2022 complaining of a painful, palpable mass in the distal third of the right femur. MRI and computed tomography (CT) scans revealed suspicious solid formations in the distal third of the femur (Figure 1). A needle biopsy confirmed the diagnosis of a GT with hemangiopericytoma‐like features. In January 2023, surgical excision was performed, followed by histological confirmation of a GT reaching the surgical margin (Figure 2). The patient was then followed regularly by a multidisciplinary team, including orthopedic surgeons, oncologists, and radiologists, with clinical assessments and imaging surveillance. No further surgical or pharmacological treatment was required during follow‐up. His past medical history was remarkable for a relevant oncological background. In 1994, a malignant mesenchymal tumor measuring 2.5 × 1.5 × 2 cm was identified in the vastus lateralis muscle of the right thigh. The patient underwent wide surgical resection followed by adjuvant chemotherapy with an adriamycin–phosphamide regimen. Histological analysis confirmed the diagnosis of a highly undifferentiated sarcoma. After several years of being symptom‐free, the patient presented with a painful mass in the right ankle. MRI performed in November 2012 revealed multiple ovoid lesions in the distal third of the right tibia, initially raising suspicion for villonodular synovitis. Consequently, an excisional biopsy was performed in 2013. Histopathological findings were consistent with a multifocal GT. Immunohistochemistry was positive for vimentin and actin, with focal positivity for H‐caldesmon and S100, confirming the diagnosis of GTs (Figure 3).

**Figure 1:**
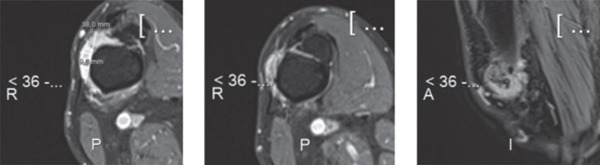
Axial and sagittal magnetic resonance imaging (MRI) of the lower limb was performed with multiplanar sequences before, during, and after intravenous paramagnetic contrast administration, revealing irregularities in the distal femoral metaphysis, with evidence of a 38 × 9 mm solid paracortical tissue exhibiting marked enhancement.

**Figure 2:**
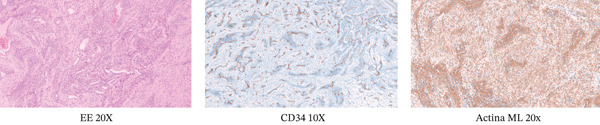
EE 20x, smooth muscle actin (Actina ML) 20x, and CD34 10x: Histology images show a well‐demarcated lesion composed of a remarkable network of thick‐walled blood vessels surrounded by a syncytial proliferation of ovoid‐to‐spindle cells. No mitotic figures, atypia, or necrosis are noted. Immunostain for smooth muscle actin shows diffuse positivity; CD34 highlights vascular endothelium.

**Figure 3:**
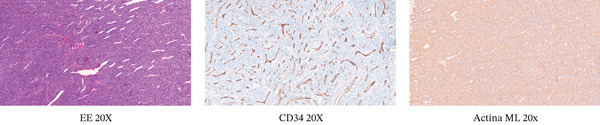
EE 20x, smooth muscle actin (Actina ML) 20x, and CD34 20x: Compared to the newly excised lesion (January 2023), previous neoplasms showed higher cellularity, with the same overall architecture and immunophenotype.

November 2025 was the patient′s most recent follow‐up visit to our institution. He is currently under oncological surveillance and remains clinically stable. The patient reports good general health, with only mild gait impairment due to the recent surgery and residual muscle weakness. The patient′s tumor history timeline is shown in Figure 4.

**Figure 4:**
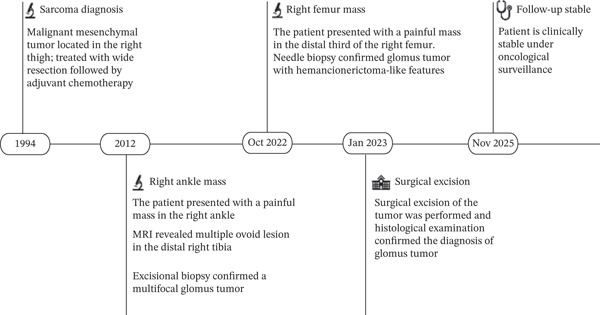
Patient′s tumor history timeline. Note: MRI, magnetic resonance imaging; Oct, October; Jan, January; Nov, November.

## 3. Materials and Methods

### 3.1. Literature Search and Study Selection

A comprehensive literature search was conducted in PubMed and Scopus between September 2025 and November 2025 using the keyword “extra digital glomus tumor,” without any restrictions on publication year. After the removal of duplicate records, titles and abstracts were independently screened to identify potentially eligible studies by two reviewers (G.D.M. and L.S.). A full‐text review was then performed to confirm study relevance and compliance with the predefined inclusion criteria. We included full‐text articles written in English reporting cases of extradigital GTs of the lower limb. Studies were excluded if the full text was unavailable, if relevant clinical or outcome data were incomplete, or when the exact anatomical location of the GT within the lower limb was not clearly specified. Figure 5 outlines the article selection process.

**Figure 5:**
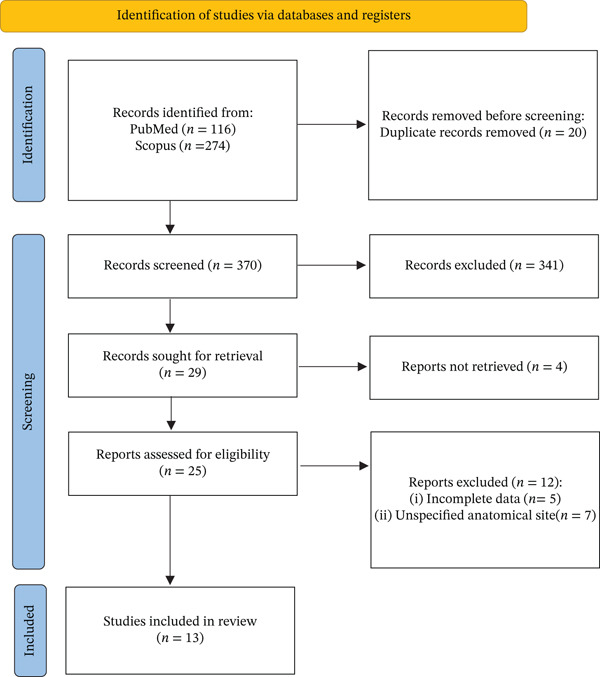
Summary of article screening and selection.

### 3.2. Data Extraction

For each eligible study, the variables collected included authors and year of publication, number of cases, patient gender, mean age, mean follow‐up, tumor location, clinical symptoms, management strategies, postoperative outcomes, and reported recurrence rates. When available, additional relevant clinical details were also recorded. In addition, for the present case, we collected detailed data on radiological characteristics based on MRI and CT findings, histopathological features, postoperative follow‐up, and the patient′s prior medical history, including any previous lesions or procedures in the same anatomical region.

### 3.3. Methodological Quality Assessment

The methodological quality of included studies was assessed by two independent reviewers (G.D.M. and G.F.P.) using the Joanna Briggs Institute (JBI) critical appraisal checklists appropriate to each study design: the JBI Checklist for Case Reports (eight items) for case reports and the JBI Checklist for Case Series (10 items) for case series [17, 18]. Each item was rated as yes, no, unclear, or not applicable. Disagreements were resolved by consensus or, when necessary, by discussion with a third reviewer (N.P.). The supporting information includes the detailed results of the methodological quality assessment performed using the JBI critical appraisal checklists for case reports and case series, reported in Tables S1 and S2.

### 3.4. Ethical Considerations and Reporting Standards

This case report was reported in accordance with the CARE guidelines, and all procedures performed in the study were in accordance with the ethical standards of the national research committee and with the 1964 Helsinki Declaration. Informed consent was obtained from the patient included in the study.

## 4. Discussion

The purpose of the present literature review was to systematically summarize the available literature concerning the extradigital GTs, with particular emphasis on lesions involving the lower limb, which represent an uncommon and often under‐recognized localization (Table 1). We identified and included 13 studies in this review that respect our predefined inclusion criteria, which mainly consisted of case reports (eight single‐patient case reports) and case series, a study design distribution that reflects both the overall rarity of GTs and the particularly uncommon occurrence of lesions in the lower extremity. The publication years of the included articles bridged a wide temporal range, from 1972 to 2024, underscoring the slow and scattered accumulation of evidence on this topic over several decades. In the current literature, we found a total of 143 patients with extradigital GTs of the lower limb, with the sample size from individual studies varying from one to 56 patients. The mean age of patients included in the studies was 45 years, with a minimum of 25 and a maximum of 65 years, and among the studies included, there were 94 male patients, accounting for more than 65% of the overall cohort. Notably, the youngest patient in the cohort, who was 25 years old, was female. We observed that these lesions tended to occur in older patients and showed a male predominance, in line with the age distribution and gender pattern reported for extradigital GTs in previous studies [10]. In our review of lower extremity extradigital GTs, the most frequently involved sites were the knee (*n* = 17), thigh (*n* = 8), foot (*n* = 6), ankle (*n* = 3), and calf (*n* = 2), whereas in some cases the exact anatomical site of the lower limb lesion was not specified. The most common reported symptoms were pain (varying from moderate discomfort to excruciating pain) and localized tenderness, followed by hyperesthesia, paresthesia, or the presence of palpable subcutaneous nodules, whereas only a minority of cases presented with limited range of motion or were clinically silent and detected incidentally. In one patient, the diagnosis was made incidentally following trauma, although the individual already exhibited a localized area of tenderness before the injury. The median follow‐up was 3 years (range from 2 months to 16 years); surgical excision was the treatment preferred, and diagnosis was confirmed by histological examination in all cases. Although four patients experienced recurrences (1‐year follow‐up) [10], patients remained symptom‐free during the follow‐up period, and complete resolution of the symptoms and no evidence of recurrence have been noted. Finally, in only two patients, persistence of symptoms was specifically reported, lasting 6 months and 2 years, respectively [11]. The patient in this case is a 64‐year‐old male who reported a painful, palpable mass in the distal third of the right femur. A needle biopsy demonstrated a GT, and a subsequent surgical excision was performed, with histological examination confirming the diagnosis. After a comprehensive review of his medical history, it emerged that he had undergone surgical excision of a malignant mesenchymal tumor of the vastus lateralis muscle of the right thigh more than 28 years earlier. Following several years without symptoms, he then developed a painful mass in the right ankle, and histopathological analysis at that time was consistent with a multifocal GT, which occurred approximately 10 years before his presentation to our institution. Compared with the newly excised lesion, the previously resected neoplasm on the right ankle showed higher cellularity with the same overall architectural pattern and immunophenotypic profile. The present case and this review contribute to the existing evidence by demonstrating that lower limb extradigital GTs tend to occur in older patients, show a male predominance, and are associated with nonspecific clinical features that can delay diagnosis for months or even years. In this context, the clinical findings observed help to refine the spectrum of clinical presentations of extradigital GTs and emphasize how important it is to consider this entity in the differential diagnosis of persistent, localized lower extremity pain or subcutaneous nodules of uncertain origin. The possible differential diagnoses of GTs include other lesions with a similar clinical or radiological appearance, such as hemangioma, neuroma, or osteochondroma. The treatment of choice is complete surgical excision, which aims to achieve full symptom relief and to prevent recurrence. Incomplete excision is considered the main cause of local tumor recurrence. Preoperative imaging can help to more accurately delineate the extent and margins of the lesion [2, 27]. To prevent misdiagnosis, immunohistochemical tests should be carried out in suspected cases [12, 29]. The patient in our study has an unusual presentation with a tumor located in the right ankle in 2012 and a new tumor in the right thigh, near the knee, in 2022. In the reviewed literature where the site of the lower limb is specified, GT localization in the thigh is present in seven cases [9, 10, 23], whereas in the ankle, it is found in three cases [9, 10]. In none of these cases is there a report of recurrence or the appearance of a new tumor during the follow‐up period, nor the presence of multiple GT localizations in the same patient. Although in this case the femoral lesion was first considered a late recurrence, the long disease‐free interval, different anatomic location, and prior multifocal disease make metachronous multifocal GT more plausible than a true recurrence. Given the rarity of extradigital GTs and the lack of molecular clonality studies, distinguishing between recurrence and separate primary lesions remains challenging. The unique aspects of our study include the extended follow‐up period, which is well beyond the average for cases reported in the literature (about 10 years following the primary GT detection in the ankle); the tumor′s location in the lower limb, making it an atypical site; and the presence of a GT in two different sites of the lower limb in the same patient, with the second tumor appearing approximately 10 years after the first. Currently, the patient is asymptomatic and in follow‐up with scheduled clinical and imaging examinations.

**Table 1:** Evidence from literature review.

Author	Case (gender)	Age (y)	Follow‐up (m)	Tumor location	Symptoms	Management	Outcomes
Temiz et al. [19]	5 (4 M, 1 F)	35	12	Lower limb (2): Crural region	Purple subcutaneous nodules with severe pain on palpation	Surgical excision	Complete resolution of the pain and no recurrence was encountered
Strahan and Bailie [11]	15 (3 M, 12 F)	39	192	Lower limb (5): Pretibial region (1), calf of leg (1), and plantar surface of foot (3)	Pain and asymptomatic (1)	Surgical excision (14)	Pain after 2 years (1) and 6 months (1)
Nguyen et al. [20]	1 (M)	32	24	Knee: Pretibial area	Persistent pain and subcutaneous mass	Surgical excision	Improvement of clinical condition and pain relief after 2 weeks; no recurrence
Lee et al. [10]	42 (33 M, 9 F)	48.26	12	Lower extremities (10): Knee (3), lower leg (3), leg (1), thigh (1), foot (1), and ankle (1)	Solitary lesions (38) and multiple skin lesions (4)	Surgical excision	Although four of these 38 patients (10.5%) experienced recurrences, most patients remained symptom‐free for 1 year
Sbai et al. [21]	1 (M)	65	12	Lower leg	Painful swelling and hyperesthesia	Surgical excision	Complete disappearance of pain without any evidence of recurrence
Schiefer et al. [9]	56 (46 M, 10 F)	59	57.6	Lower extremity (23): Buttock (1), thigh (5), knee (10), leg (3), ankle (2), and foot (2)	Pain and localized tenderness	Complete excision (38), punch biopsy (15), and shave biopsy (3)	48 patients were symptom‐free after the removal of their tumors without recurrence
Granel et al. [22]	1 (F)	54	12	Calf	Pain, tenderness, and paresthesia with burning sensations	Surgical excision	Complete resolution of the symptoms, no evidence of recurrence has been noted
Sbai et al. [23]	1 (F)	25	12	Thigh	Pain and hyperesthesia, exacerbated by movements	Surgical excision	Relieved from pain
Gonçalves et al. [24]	1 (M)	51	12	Knee	Accidental diagnosis after trauma and tender area on the lateral aspect of the knee	Surgical excision	Complete remission of the symptoms
Amillo et al. [25]	1 (F)	38	12	Vastus lateralis	Pain and limited knee extension	Surgical excision	Immediate relief of pain, no recurrence
González‐Llanos et al. [26]	1 (M)	50	NR	Lateral surface of the lower metaphysis of the femur, near the knee	Tenderness	Surgical excision	Relieved from pain
Chou et al. [27]	17 (3 M, 14 F)	47.4	NR	Lower extremity (7)	Pain and tenderness	Surgical excision	Complete symptom relief
Chouairy et al. [28]	1 (M)	49	2	Thigh	Pain and localized tenderness	Surgical excision	Relieved from pain

Abbreviations: F, female; M, male; m, months; NR, not reported; y, year.

## 5. Conclusions

There are no similar cases reported in the literature, characterized by the presence of an extradigital GT in an uncommon site (the lower limb) and the appearance of a new tumor after approximately 10 years.

## Funding

No funding was received for this manuscript. Open access publishing facilitated by Universita Campus Bio‐Medico di Roma, as part of the Wiley ‐ CRUI‐CARE agreement.

## Ethics Statement

All procedures performed in the study were in accordance with the ethical standards of the national research committee and with the 1964 Helsinki Declaration.

## Consent

Informed consent was obtained from all individual participants included in the study.

## Conflicts of Interest

The authors declare no conflicts of interest.

## Supporting information


**Supporting Information** Additional supporting information can be found online in the Supporting Information section. Table S1: Quality of papers included according to the Joanna Briggs Institute critical appraisal checklists for case reports. Table S2: Quality of papers included according to the Joanna Briggs Institute critical appraisal checklists for case series.

## Data Availability

The data that support the findings of this study are available upon request from the corresponding author. The data are not publicly available due to privacy or ethical restrictions.
