# Supplementary material for: Metachronous Multifocal Extradigital Glomus Tumor of Lower Extremity: A Case Report and Literature Review
Source: Case Rep Orthop. 2026 Jul 24;2026:1411715. doi: 10.1155/cro/1411715 (PMC13397867; doi:10.1155/cro/1411715)
Supplement: Supplementary file 1 — Supporting Information Additional supporting information can be found online in the Supporting Information section. Table S1: Quality of papers included according to the Joanna Briggs Institute critical appraisal checklists for case reports. Table S2: Quality of papers included according to the Joanna Briggs Institute critical appraisal checklists for case series. [file CRO-2026-1411715-s001.docx]

| **Article** | Q1: Were patient’s demographic characteristics clearly described? | Q2: Was the patient’s history clearly described and presented as a timeline? | Q3: Was the current clinical condition of the patient on presentation clearly described? | Q4: Were diagnostic tests or assessment methods and the results clearly described? | Q5: Was the intervention(s) or treatment procedure(s) clearly described? | Q6: Was the post-intervention clinical condition clearly described? | Q7: Were adverse events (harms) or unanticipated events identified and described? | Q8: Does the case report provide takeaway lessons? |
| --- | --- | --- | --- | --- | --- | --- | --- | --- |
| Gonçalves et al. 2014 [24] | Yes | Yes | Yes | Yes | Yes | Yes | No | Yes |
| Chouairy et al. 2024 [28] | Yes | Yes | Yes | Yes | Yes | Yes | No | Yes |
| Sbai et al. 2018 [21] | Yes | Yes | Yes | Yes | Yes | Yes | No | Yes |
| Granel et al. 2013 [22] | Unclear | Unclear | Yes | Unclear | Yes | Unclear | Unclear | Yes |
| Sbai et al. 2018 [23] | Yes | Yes | Yes | Yes | Yes | Yes | No | Yes |
| González-Llanos et al. 2000 [26] | Yes | Yes | Yes | Yes | Yes | Yes | No | Yes |
| Amillo et al. 1997 [25] | Yes | Yes | Yes | Yes | Yes | Yes | No | Unclear |
| Nguyen et al. 2021 [20] | Yes | Yes | Yes | Yes | Yes | Yes | No | Yes |

**Supplementary Table I** – Quality of papers included according to the JBI checklist. (1)

| Article | Q1: Were there clear criteria for inclusion in the case series? | Q2: Was the condition measured in a standard, reliable way for all participants included in the case series? | Q3: Were valid methods used for identification of the condition for all participants included in the case series? | Q4: Did the case series have consecutive inclusion of participants? | Q5: Did the case series have complete inclusion of participants? | Q6: Was there clear reporting of the demographics of the participants in the study? | Q7: Was there clear reporting of clinical information of the participants? | Q8: Were the outcomes or follow up results of cases clearly reported? | Q9: Was there clear reporting of the presenting site(s)/clinic(s) demographic information? | Q10: Was statistical analysis appropriate? |
| --- | --- | --- | --- | --- | --- | --- | --- | --- | --- | --- |
| Schiefer et al. 2006 [9] | Yes | Yes | Yes | Unclear | Yes | Yes | Yes | Yes | Yes | Yes |
| Chou et al. 2016 [27] | Yes | Yes | Yes | Unclear | Yes | Yes | Yes | Yes | Unclear | No |
| Lee et al. 2011 [10] | Yes | Yes | Yes | Unclear | Unclear | Yes | Yes | Yes | Yes | No |
| Strahan et al. 1972 [11] | Yes | Unclear | Yes | Unclear | Unclear | Yes | Yes | Yes | Unclear | No |
| Temiz et al. 2016 [19] | Yes | Yes | Yes | Unclear | Yes | Yes | Yes | Yes | Yes | No |

**Supplementary Table II** – Quality of papers included according to the JBI checklist (2).

1. Moola S, Munn Z, Tufanaru C, Aromataris E, Sears K, Sfetcu R, Currie M, Qureshi R, Mattis, P, Lisy K, Mu P-F. Joanna Briggs Institute Reviewer’s Manual. 2017th edn. In. p. Chapter 7: Systematic reviews of etiology and risk. Available from: https://reviewersmanual.joannabriggs.org/

2. Munn Z, Barker TH, Moola S, Tufanaru C, Stern C, McArthur A, et al. Methodological quality of case series studies: an introduction to the JBI critical appraisal tool. JBI Evid Synth. 2020 Oct;18(10):2127–33. doi:10.11124/JBISRIR-D-19-00099 PubMed PMID: 33038125.
